# Effectiveness of a Blend of Pelvic Proprioceptive Neuromuscular Facilitation, Task-Oriented Approach, and Rood’s Approach in a Three-Year-Old Child With Spastic Diplegia: A Case Report

**DOI:** 10.7759/cureus.31063

**Published:** 2022-11-03

**Authors:** Pranali M Pachkhede, Vikrant G Salphale, Pooja Dhage, Nikita S Deshmukh

**Affiliations:** 1 Physiotherapy, Ravi Nair Physiotherapy College, Datta Meghe Institute of Medical Science, Wardha, IND; 2 Neuro Physiotherapy, Ravi Nair Physiotherapy College, Datta Meghe Institute of Medical Sciences, Wardha, IND; 3 Musculoskeletal Physiotherapy, Ravi Nair Physiotherapy College, Datta Meghe Institute of Medical Sciences, Wardha, IND

**Keywords:** proprioception neuromuscular facilitation, task-oriented approach, rood’s approach, cerebral palsy, spastic diplegia

## Abstract

As a result of non-progressive brain damage, cerebral palsy (CP) has traditionally been seen as a disorder of movement and posture; however, more recent classifications enable clinicians to understand more than just the movement issue. Research has evolved with the accurate categorization of cerebral palsy into distribution, motor type, and functional level. Children with spastic diplegia usually have pelvic asymmetry, which affects the child's functional abilities, including their ability to balance and walk independently. Physical therapists currently treat this illness using a variety of treatments, each of which is significant in its own way. A model for enhancing organizational capabilities is clinical management in physical therapy, which incorporates effective practices supported by research and improves outcomes. This case study demonstrates the efficiency of a deliberate physical therapy strategy to enhance functional independence in a three-year-old male child with spastic diplegia. The young patient complained of difficulties with balance and toe-walking and a delay in reaching age-appropriate milestones when seen in the neuro physiotherapy outpatient department. History demonstrated that a delayed cry occurred with an abrupt onset of fever, foaming at the mouth, and other symptoms described.

## Introduction

Cerebral palsy (CP) is a condition brought on by a non-progressive brain injury. The prevalence of cerebral palsy varies widely, from 1.5 to 3 children per 1000 people [1,2]. Its motor abnormalities are frequently followed by secondary musculoskeletal issues, seizures, and impairments in sensation, perception, cognition, communication, and behaviour [3]. Children who are premature frequently have developmental impairments. Spastic, dyskinetic, and ataxic cerebral palsy are the three clinical subgroups of the disorder, depending on the predominant motor impairment. Developmental delay refers to any delay in a child reaching milestones suitable for their age. The most prevalent type of CP that causes issues with posture, balance, and gait control is spastic diplegia [4]. One feature of upper motor neuron syndrome that can impair a child's functionality, limit everyday activities, and lower their quality of life is spasticity [5]. Spastic diplegia, the most prevalent type of CP, has a major influence on functional performance and gait and mostly affects the lower limbs [6]. Children with diplegic CP frequently experience delayed gross motor function development, which has been linked to functional outcomes, including activities of daily living. [7]. Due to calf muscle stiffness, standing and walking are delayed, resulting in the ankles being in an equino-varus position. Growing older can result in hip flexor and hamstring tightness, which makes extended walking challenging and gives the appearance of neurological deterioration [8]. Children with diplegic CP frequently experience delayed gross motor function development, which has been linked to functional outcomes, including activities of daily living [9].

For children with CP, there are several therapy options available. To improve balance and posture, the therapist should also facilitate effective trunk extension and increased trunk and pelvic mobility [1]. A paradigm for improving organisational capability, including evidence-based best practices, and improving results is clinical management in physical therapy. The main risk factors for CP include low birth weight, uterine disorders, and numerous pregnancies. CP is four to six times more likely to affect underweight newborns. For full-term newborns and preterm infants, intrauterine infections increase the risk of cerebral palsy by five times and two times, respectively [2]. There are several therapeutic choices available for these people. The majority of these techniques work well together, and none of them is mutually incompatible. For the best results, a variety of modalities must be employed to treat children with dystonic cerebral palsy because there is no "magic bullet" [10].

## Case presentation

Patient Information

A three-year-old male baby was taken to the outpatient department after a routine vaginal birth and had a history of fever, drowsiness, and mouth drooling eight months earlier. He experienced a general delay in development and was delivered at term without any postpartum complications. On the third day after delivery, the patient was brought to the local hospital with a fever and difficulty feeding; however, no anomaly was detected, allowing for discharge with routine follow-up. The patient was last inoculated against pulse polio at 1.5 years of age.

As narrated by the mother, the patient had three episodes of convulsions, and the reason for the three episodes was fever. In one episode, the child showed rolling eyes. The first episode was when the patient was three days old, the second episode was when the patient was six months old, and the third episode was when the patient was two years old. Postictal sleepiness and unconsciousness followed the 25- to 30-minute long session. No loss of consciousness or abnormal behaviour was seen. He did not mention any discharge, nosebleeds, colds, vomiting, or loose stools in his medical history. The child was once again brought to a local hospital after one and a half months with complaints of fever and sleepiness. He was treated with drugs including phenytoin, baclofen, and carbamazepine. After three days in the hospital, he was discharged on the doctors' recommendations. He was conscious, afebrile, and had stable vital signs when he was discharged. The mother saw that the infant was having trouble transitioning from one activity to another and was unable to sit independently. The child was tested last month because he had not yet learned to stand independently at the age of one. The child was sent to the hospital for further treatment.

Clinical findings

The infant was ectomorphic in appearance, aware and cooperative, and well-oriented to time, place, and person. The right ankle was found to be in plantar flexion upon inspection. There was a tongue tie. When examined, the hamstrings had more tone, a grade of 1+ on the modified Ashworth scale. According to paediatric muscle testing, the upper limbs have higher muscular power (grade 4) than the lower limbs (grade 3). On the lower leg, deep tendon reflexes were exaggerated (Table 1 shows the grading of muscle tone according to the Modified Ashworth Scale), and a plantar extensor was seen upon admission. On both lower limbs, the hamstrings, adductors, and calf muscles were all tight (Table 2 shows deep tendon reflexes). The baby can crawl and transition from being supine to sitting with decent static balance, but their dynamic seated balance is compromised, and they have trouble standing and walking on their own.

**Table 1:** Grading of muscle tone according to Modified Ashworth Scale

Tone	On assessment	After 2 months
Hip flexor	Grade 1	Grade 0
Hip extensor	Grade 1+	Grade 1
Knee flexor	Grade 1	Grade 0
Knee extensor	Grade 1	Grade 0
Ankle plantar-flexion	Grade 2	Grade 1
Ankle dorsiflexion	Grade 2	Grade 1
Ankle inversion	Grade 2	Grade 1
Ankle eversion	Grade 2	Grade 1

**Table 2:** Deep tendon reflexes

Deep tendon reflexes	Grading	Result
Patellar tendon/Knee jerk	3+	Brisker than average, slightly hyper-reflexic
Achilles tendon jerk	3+	Brisker than average, somewhat hyper-reflexic

Outcome measures

Gross motor skills include balance skills, and poor balance makes it difficult to do functional daily activities. Accurate, reliable, and simple assessments of functional balance are crucial in paediatric clinical settings because balance is associated with functional activity. (i) The 14-item Paediatric Balance Scale (PBS) assesses functional balance in everyday tasks. It is a criterion-referenced test. Each item receives a 4-point rating. The PBS, a modified version of the Berg Balance Scale (BBS), was developed as a balance measure for children with mild to severe motor impairments and has good test-retest and inter-rater reliability [11]. (ii) To evaluate trunk dysfunction, the static sitting balance (TIS-SSB), dynamic sitting balance (TIS-DSB), and trunk coordination (TIS-C) subcomponents of the Trunk Impairment Scale are used. The 17 elements that make up the TIS have three subtotal scores that range from 0 (poor performance) to 23 (excellent performance) (best performance) [12]. (iii) The Gross Motor Function Measure Scale (GMFCS) categorises a child's present gross motor function objectively. The child's self-initiated mobility is the main focus, with a focus on sitting and walking functions in particular. Children in level I have the greatest autonomous motor function, while those in level V have the least. The function is divided into five levels [13]. (iv) The Modified Ashworth Scale, which uses a 5-point numerical rating system to classify spasticity, ranges from 0 to 4, with 4 denoting limb rigidity in flexion or extension [14]. It has been found that the MAS's inter-rater reliability for determining the degree of spasticity is variable [15].

Physiotherapy functional assessment

Both limbs' strength was rated favourably on a qualitative scale; nevertheless, the lower limbs scored poorly to fairly. He experienced hamstring, short-adductor, and bilateral Achilles tendon discomfort. He had difficulty walking and moving about on his own. The patient has a scissor gait and walks on his toes when forced to stand and walk with maximal assistance. The MAS grade for hamstrings on the first testing day was 1+. This child had a PBS score of 43/56, a Trunk Impairment Scale score of 10/23, and a GMFCS stage of III.

Physiotherapeutic interventions

**Figure 1:**
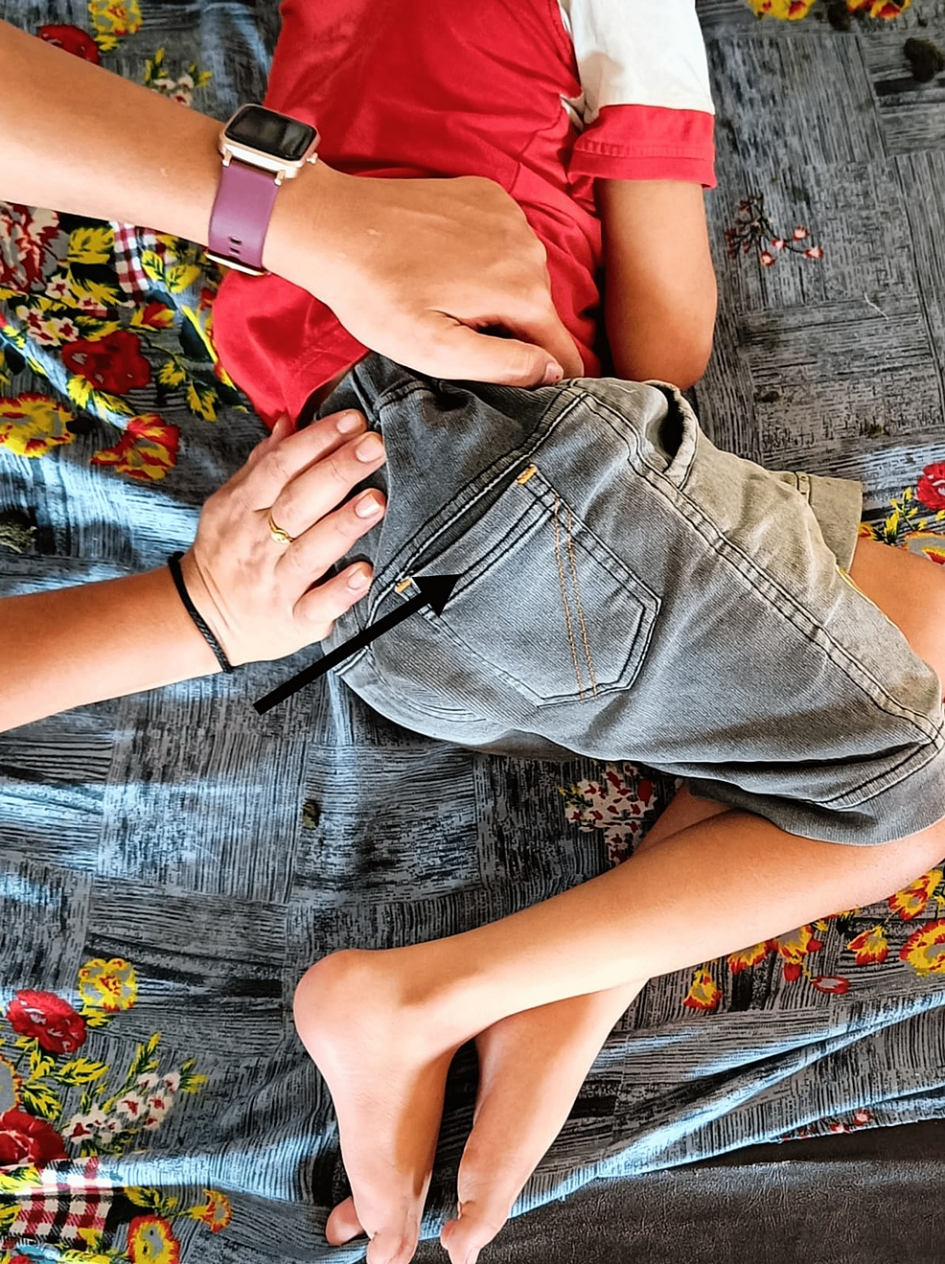
Delivering pelvic proprioceptive neuromuscular facilitation to the child in a side lying

**Figure 2:**
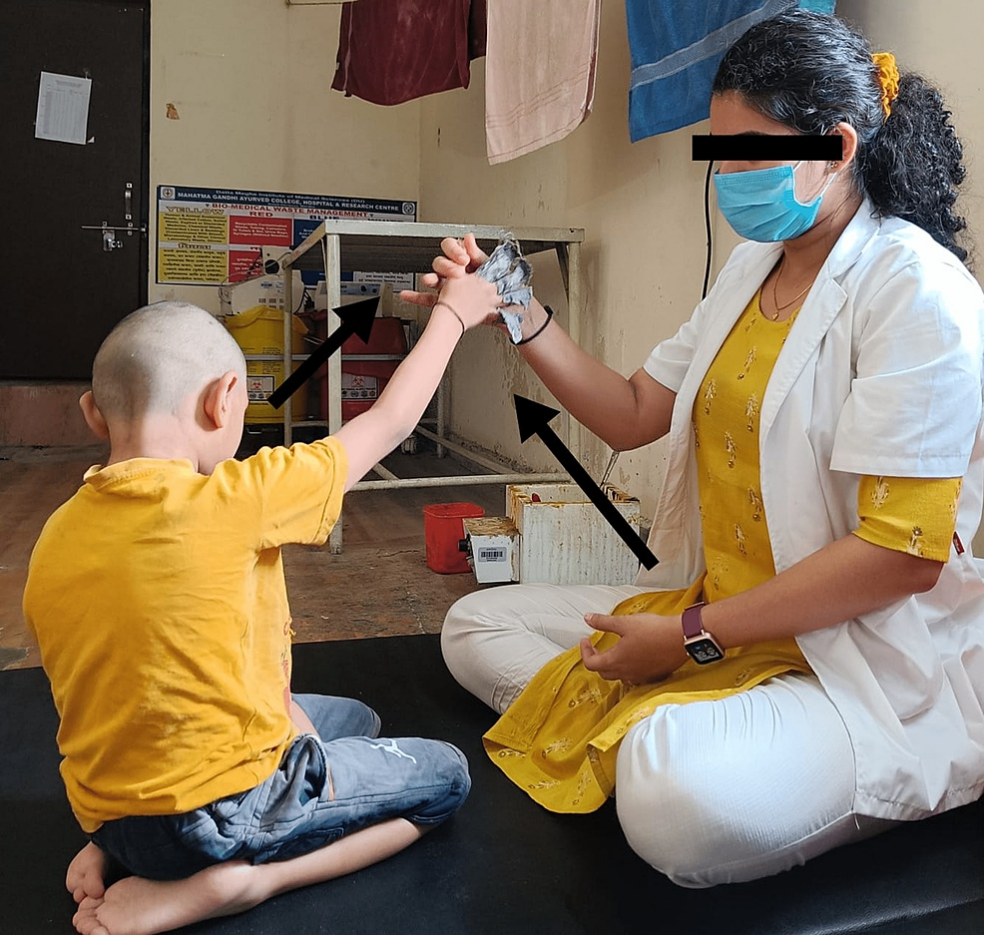
Reach out activities to the child in kneeling position

**Figure 3:**
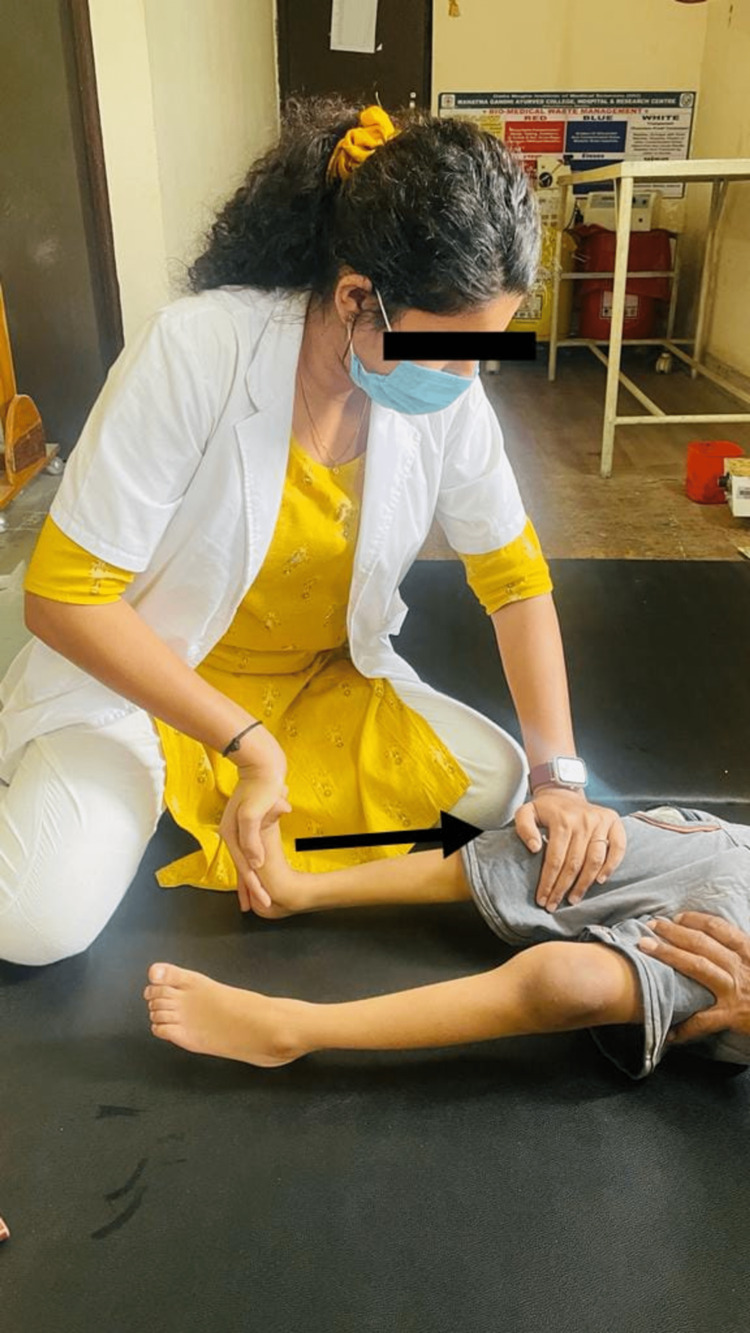
Prolonged stretching of calf muscle

**Figure 4:**
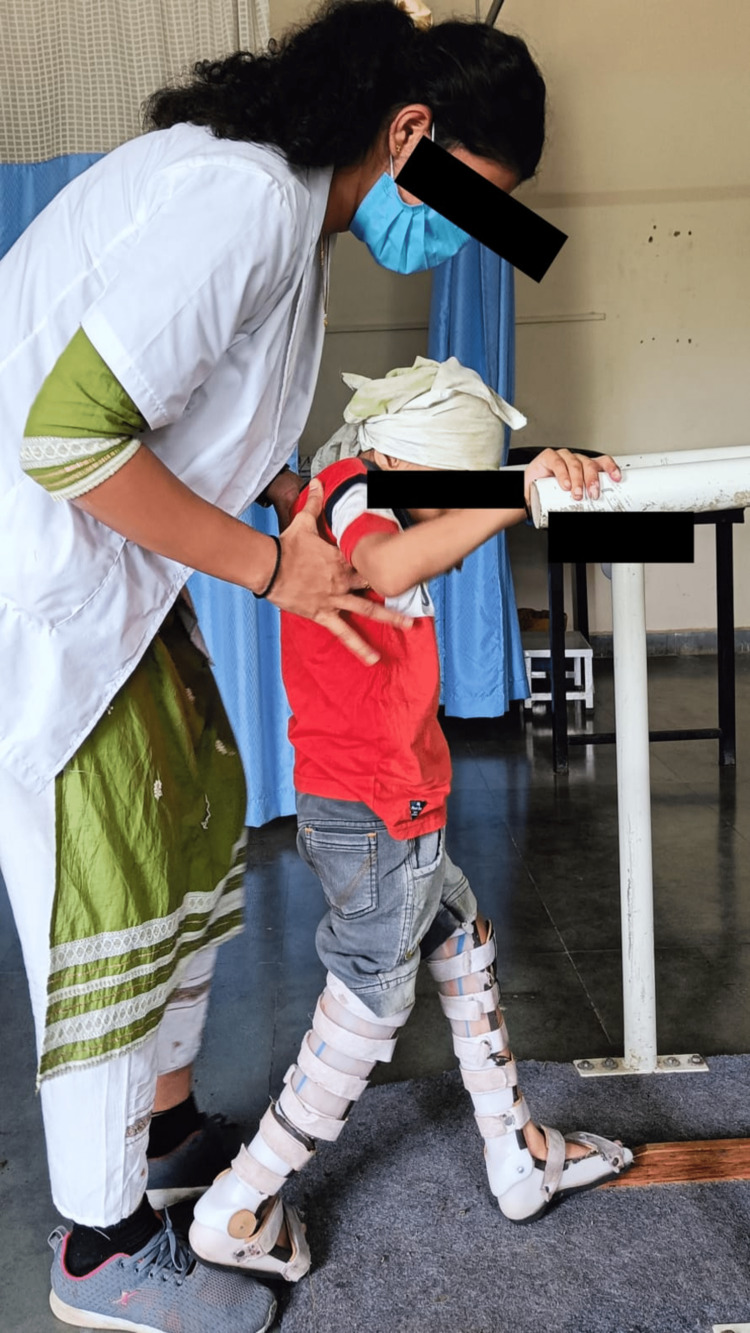
Gait training with involvement of mirror biofeedback

Physiotherapy implementations can prevent contractures with the use of passive, mild range-of-motion exercises and stretches throughout the main joints. Consistently scheduling resistive workouts that target all of the main muscle groups with low resistance and plenty of repetitions increases local muscular endurance. Exercises for physiotherapy are meant to help with mobility and transfers while also enhancing balance, postural control, and gait. Treatment goals include correcting the child’s pelvic mal-alignment and improving their balance and walking skills. Table 3 shows a description of the intervention strategies implemented with the child. Figure 1 shows the delivery of pelvic proprioceptive neuromuscular facilitation (PNF) to the child in a side-lying position. In Figure 2, reach-out activities for the child in a kneeling position are depicted. Figure 3 shows the prolonged stretching of the calf muscle. In Figure 4, gait training with the involvement of mirror biofeedback is shown.

**Table 3:** Description of the intervention strategies implemented in the child PNF: proprioceptive neuromuscular facilitation

Problem identified	Problem cause	Goal framed	Physiotherapy interventions
Lack of involvement of the child in a therapy session	The low span of attention	To engage the child in the therapy session	Play therapy activities with a ball for a span of 15 minutes
Pelvic mal-alignment	Spasticity	To restore normal pelvic congruency	PNF techniques for inferior limbs. Rhythmic initiation and rhythmic stabilization for a span of 20 minutes on both sides (Figure 1).
Musculoskeletal defacements	The inactive base of support	To normalize balance and gait parameters	Task-oriented approach-reach out activities to the child in a kneeling position to improve the dynamic balance. 5-7 repetitions on a single side for a span of 15 minutes (Figure 2).
Spasticity of hamstrings, adductors and tendo-achillis	Spasticity	To normalize the muscle tone in hypertonic muscle groups	Rood's inhibitory techniques like deep tendinous pressure, prolonged icing for 20 minutes, sustained stretching of hamstring, adductors and tendo-achillis for 30 seconds with three rep (Figure 3).
Difficulty waking independently	Inadequate trunk control	To maintain the functional position of the ankle while walking	Gait training in parallel bars with an AFO in front of a mirror to gain feedback from it (Figure 4).

Follow-up and outcome

After six weeks of integrated neuro-physiotherapy procedures that comprised passive stretching and task-oriented strategies, the child had very good proximal stability. By the end of four weeks, the spasticity had decreased and the tone had returned to near normal. Although he made transitions voluntarily, there were some uncontrolled movements as he attempted to assume the opisthotonus position. Therefore, to overcome these problems, kneeling to quadruped and kneeling to half-kneeling transitions were included. The infant was able to walk with just minimal assistance from the caregiver after making good initial contact. The baby's prognosis strategy is to transition during the next six weeks with less unwanted activity and more intentional movement. The post-intervention outcomes displayed a significant improvement in the child. The scoring on the Paediatric Balance Scale was 50/56, the Trunk Impairment Scale was 17/23, the Gross Motor Function Classification System was stage II, and the Modified Ashworth Scale was grade 1 for both inferior limbs.

## Discussion

The child's clinical characteristics met up with current research on spastic cerebral palsy. The Modified Ashworth scale, Paediatric Balance Scale, Trunk Impairment Scale, and Gross Motor Function Measure Scale were used to assess the effectiveness of the integrated physiotherapy management, which was created using play therapy, pelvic PNF, task-oriented approach, Rood’s approach, and gait training with AFO.

The child responded well to the therapy. He had excellent session compliance, which we credit to the kind of treatment strategy and techniques used, which included entertaining tasks throughout the sessions. That study and our therapeutic strategy are complementary. The caregiver's constancy in providing home exercise routines and holding several sessions to allay the mother's concerns is also attributed to the effectiveness of the treatment programme [16]. We saw consistent improvement week after week. After a month of therapy, the kid was able to stand with the help of a sitting posture, maintain a sitting balance, and begin reaching out. The little child was walking with little assistance and had good trunk and pelvic control after six weeks, much to the delight of the caregivers. The results of a study indicate that improving postural control and balance through an eight-week neurodevelopmental therapy centred on posture and balance training is an effective way to raise the functional motor level and functional independence in spastic CP [17]. A long-term follow-up was done to go through how physical therapy affects the development of fine motor abilities.

The strength of this study is that the trio of interventions was efficient in rectifying the functional deficits in a child with spastic diplegia. Also, the limitation is the lack of uniformity in the child's cooperation during the therapy session.

## Conclusions

According to this case study, infants with spastic CP benefit from early integrative neuro-physiotherapy in combination with a goal-oriented treatment regimen that includes task-oriented approaches. In terms of the outcome metrics as well as clinically, the child improved. The results of this case study showed the benefit of providing an organised and timed physiotherapy regimen in restoring functional impairments in a child with spastic diplegia. The child improved on the Modified Ashworth Scale for lower limbs to grade 1, the Paediatric Balance Scale to 50/56, and the GMFCS to level II. The child was able to walk with little assistance after a month, and after two months, he was able to walk without assistance. He was able to control his trunk and pelvis after therapy.
